# ﻿A new species of the genus *Glossobalanus* (Hemichordata, Enteropneusta, Ptychoderidae) from China

**DOI:** 10.3897/zookeys.1202.111852

**Published:** 2024-05-27

**Authors:** Xianan Fu, Weijian Guo, Zhongwen Ding, Wenliang Zhou, Fuwen Wei

**Affiliations:** 1 Center for Evolution and Conservation Biology, Southern Marine Science and Engineering Guangdong Laboratory (Guangzhou), Guangzhou, 511458, China Center for Evolution and Conservation Biology, Southern Marine Science and Engineering Guangdong Laboratory Guangzhou China; 2 CAS Key Laboratory of Animal Ecology and Conservation Biology, Institute of Zoology, Chinese Academy of Sciences, Beijing, 100101, China Institute of Zoology, Chinese Academy of Sciences Beijing China; 3 College of Forestry, Jiangxi Agricultural University, Nanchang, 330045, China Jiangxi Agricultural University Nanchang China

**Keywords:** Conservation, external morphology, Hainan, molecular analysis, morphometry

## Abstract

A morphological and molecular analyses of a newly discovered species, *Glossobalanusweii***sp. nov.**, from Danzhou city, Hainan Island, China is presented. Several morphological characters distinguish this new species, while molecular analyses confirm significant genetic divergence from its recognized congeners (p-distance > 0.25 in mitochondrial genomes). Phylogenetic analyses place the new species in a distinct sister clade to *G.polybranchioporus*, which is afforded first-class state protection in China. An updated retrieval table is provided for the eight species of Hemichordata found in China. Hemichordate diversity remains underestimated and this new species emphasizes the need for their ongoing conservation in southern China.

## ﻿Introduction

While Hemichordata now belong to the Ambulacraria clade on a par with Echinodermata, they are nevertheless considered the closest relatives to chordates, because they typically share common features such as bilateral symmetry, gill slits and early axial patterning. Hemichordates occupy an important position in the study of the origin and evolution of deuterostomes. The phylum Hemichordata comprises approximately 130 living species worldwide (Tassia et al. 2016), divided into two classes: Enteropneusta (commonly known as acorn worms) and recently the Graptolithoidea (sea angels) (Swalla and van der Land 2024). The extant and extinct taxa of the Enteropneusta was well reviewed in Maletz (2023). The newly accepted class Graptolithoidea embraces two taxa: Graptolithina and Pterobranchia. The Enteropneusta is further classified into four families: Harrimaniidae Spengel, 1901, Ptychoderidae Spengel, 1893, Spengelidae Willey, 1898, and Torquaratoridae Holland, Clague, Gordon, Gebruk, Pawson & Vecchione, 2005. The family Saxipendiidae is placed within Harrimaniidae according to the Hemichordata World Database (Deland et al. 2010). The Ptychoderidae are characterized by a highly developed branchiogenital region and hepatic sacculations.

**Figure 1.**
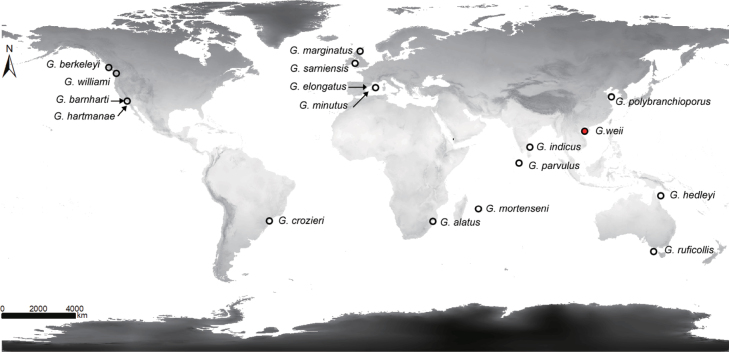
The global distribution of all *Glossobalanus* species. The distributions were compiled from the original description data. The type locality of *Glossobalanusweii* sp. nov. on Hainan Island, China was marked in red.

Seven species of hemichordates are recorded from China: *Glossobalanuspolybranchioporus* Tchang & Liang, 1965; *Saccoglossushwangtauensis* Tchang & Koo, 1935; *Balanoglossusmisakiensis* Kuwano, 1902; *B.carnosus* Müller in Spengel, 1893; *Glossobalanusmortenseni* van der Horst, 1932; *Ptychoderaflava* Eschscholtz, 1825, and *Glandicepsqingdaoensis* An & Li, 2005. In China, *G.polybranchioporus* and *S.hwangtauensis* are under first-class state protection and the other five species are under second-class state protection as rare and endangered wildlife. Despite the wide distributional range of the known sixteen species of *Glossobalanus*, from European Seas to the northeast Pacific, many regions of their biogeographic range remain unexplored (Fig. 1).

During recent field surveys along the Chinese coast, we discovered specimens of the genus *Glossobalanus* from Toudong village in Danzhou City, Hainan Province (Fig. 1), which were not previously known. Detailed comparisons with all known *Glossobalanus* species revealed significant morphological differences between this new record and its relatives, particularly in proboscis and collar morphology. Further genetic analyses confirmed its phylogenetic distinctiveness from currently recognized congeners. Here we describe this new species as *Glossobalanusweii* sp. nov. and provide an updated key to identify the different species of hemichordates in China. We also discuss their diversity and conservation issues.

## ﻿Materials and methods

### ﻿Taxonomic sampling

Specimens of *Glossobalanusweii* sp. nov. were collected from Toudong village, Danzhou, Hainan Province, China in February and July 2023. Two specimens for histological investigation were fixed in Bouin’s solution and dehydrated in a graded series of ethanol. The voucher specimens are stored in 70% ethanol at the Southern Marine Science and Engineering Guangdong Laboratory (Guangzhou) (also named Guangzhou Marine Laboratory, for short GML) Biological Resource Sample Bank. Specimens for comparison of *Glossobalanuspolybranchioporus*, *Balanoglossusmisakiensis*, and *Glandicepsqingdaoensis* were donated by Professor Xinzheng Li of the Institute of Oceanology, Chinese Academy of Sciences, collected from Jiaozhou Bay, Qingdao, Shandong Province, China. Fresh trunk tissue of these specimens was used for comparative genetic analysis.

### ﻿Morphological data

Total length (**ToL**), proboscis length (**proL**), collar length (**colL**), hepatic region length (**hepL**), branchial length (**brL**) (from the beginning of branchiogenital region to the end of the gill region), and branchiogenital region length (**brgL**) of the specimens were measured using ImageJ2. Length analysis was performed three times and the average value was taken. For older papers that do have relevant length records, measurements were taken from pictures that included a scale. The specimens for histology investigation were cut into frozen sections (10 μm thickness) after being submerged in optimal cutting temperature compound (OCT). Thin sections from the largest frozen sections were selected for hematoxylin and eosin (HE) staining. Thin sections were viewed and photographed with a Nikon, Eclipse Ti2 photomicroscope. All materials examined are deposited in GML.

### ﻿Genetic data

Molecular genomic DNA was extracted from the trunk of four specimens using the SDS method followed by purification with QIAGEN® Genomic kit (Cat#13343, QIAGEN), according to the standard operating procedure provided by the manufacturer, and was sequenced using the MGI-DNBseqT7 platform. Following the MitoZ tutorial (Meng et al. 2019), a total of 5 G base pairs (bp) of each specimen were obtained respectively, and their complete mitochondrial genome was generated. The assembled mitochondrial genomes were automatically annotated and manually corrected in Geneious Prime 2022.2.2. The mitogenome sequence assembly N_001485032, N_001485033, N_001485034 and N_001485035 were uploaded to China National GeneBank (https://db.cngb.org/). In addition, the available mitochondrial genome sequences of congeners were downloaded from GenBank (https://www.ncbi.nlm.nih.gov/genbank/). Available accessions are listed in Table 1.

**Table 1.** Mitochondrial genome sequences used in this study.

Family	Species	Accession No.
Cephalodiscidae	*Cephalodiscushodgsoni* Ridewood, 1907	MF374802
Harrimaniidae	*Saccoglossuskowalevskii* (Agassiz, 1873)	NC_007438
Harrimaniidae	*Stereobalanuscanadensis* (Spengel, 1893)	NC_040107
Ptychoderidae	*Balanoglossuscarnosus* Müller in Spengel, 1893	NC_001887
Ptychoderidae	*Balanoglossusclavigerus* Delle Chiaje, 1829	NC_013877
Ptychoderidae	*Balanoglossusmisakiensis* Kuwano, 1902	N_001485032
Ptychoderidae	*Glossobalanusmarginatus* Meek, 1922	NC_040109
Ptychoderidae	*Glossobalanuspolybranchioporus* Tchang & Liang, 1965	N_001485033
Ptychoderidae	*Glossobalanusweii* sp. nov.	N_001485035
Rhabdopleuridae	*Rhabdopleuracompacta* Hincks, 1880	FN908482
Spengelidae	*Schizocardiumbrasiliense* Spengel, 1893	NC_040108
Spengelidae	*Glandicepsqingdaoensis* An & Li, 2005	N_001485034

Species names were checked on WoRMS (www.marinespecies.org). Mitochondrial genome accessions were from GenBank (https://www.ncbi.nlm.nih.gov/genbank/) and China National GeneBank (https://db.cngb.org/).

### ﻿Phylogenetic analyses

Sequences were aligned using MAFFT (Kuraku et al. 2013) with the default parameters and further trimmed by trimAl (Capella-Gutiérrez et al. 2009) with parameters -gt 0.6. The aligned DNA sequences of 13 protein-coding genes in the mitochondrion were concatenated in a single dataset (total 11,281 bp) and subjected to Bayesian inference (BI) analyses under the optimal partitioning scheme. The genetic data were partitioned into five unequivocal data blocks and the best-fit nucleotide substitution model for each block was identified using the PartitionFinder v. 2.1.1 (Lanfear, et al. 2017) which nesting in IQ-TREE v. 1.6.12 (Nguyen et al. 2015). The nucleotide models GTR+I+G or GTR +G were used in our analysis, considering the constraints of the available models in MrBayes (Ronquist et al. 2012) and the recommendations provided by PartitionFinder. BI analyses were performed using MrBayes v. 3.2. Two independent Markov chain Monte Carlo (MCMC) runs were performed with three heated and one cold chain for 10^6^ generations and sampled every 1,000 generations. To judge convergence and effective sample sizes (ESS) for all parameters, Tracer v. 1.7 (Rambaut et al. 2018) was used to analyze, all the ESS values exceeding 100 after the first 25% generations were discarded. The consensus tree and posterior probabilities were estimated from the remaining generations of two runs and compared for congruence.

## ﻿Results

### ﻿Morphological results

The specimens were morphologically consistent with the recognized species from the genus *Glossobalanus*. They differ from the other 16 species of *Glossobalanus* in their combination of morphological characters, including larger size, and proboscis to collar proportions and markings (Table 2). The taxonomic account is as follows.

**Table 2.** Morphometric measurements and distribution of *G.weii* sp. nov. and all other sixteen species of *Glossobalanus*.

Species	ToL (mm)	proL (mm)	colL (mm)	hepL (mm)	proL/colL	brgL/ brL
*G.alatus* van der Horst, 1940	34.5	2	3	3.7	<1	9 –10
*G.barnharti* * Cameron & Ostiguy, 2013	67	2.1 –2.4	1.7 –2.1	14.8	1 –1.4	3
*G.berkeleyi* * Willey, 1931	60.7	4.91	3,39	–	1.4	4
*G.crozieri^+^* van der Horst, 1924	30 –50	2.5 –4	3	–	0.8 –1.3	2
*G.elongatus* Spengel, 1904	–	–	–	–	–	–
*G.hartmanae* Cameron & Ostiguy, 2013	23 –45	3.5	3.5	4	1	4.5
*G.hedleyi* (Hill, 1897)	–	–	–	–	–	–
*G.indicus* Rao, 1955	–	–	–	–	–	–
*G.marginatus* Meek,1922	50	7	5	–	1.4	3 –4
G.minutus* (Kowalevsky, 1866)	42	3	2	–	1.5	2 –3
*G.mortenseni* van der Horst, 1932	38.5 –67	4.5 –4.9	3.2 –3.7	4.9 –7.6	2 –2.4	–
*G.parvulus* Punnett, 1906	–	–	–	–	–	–
*G.polybranchioporus* Tchang & Liang, 1965	352 –613	10 –12	8 –10	40 –74	1 –1.4	4 –6
*G.ruficollis* (Willey, 1899)	–	4 –4.5	6.5 –7	–	<1	9 –10
*G.sarniensis* (Koehler, 1886)	35 –100	–	–	–	~1	6 –9
*G.williami* Cameron & Ostiguy, 2013	130	2.5	1.5	–	1.7	–
*G.weii* sp. nov.	176 –196	11.1 –15.9	5.2 –6.6	24 –26.2	2.1 –2.4	5

*Measured according to the picture with standard scale. *^+^G.crozieri* was based on description in Petersen and Ditadi (1971). Abbreviations: branchiogenital region length (brgL); branchial length (brL); collar length (colL); hepatic region (hepL); proboscis length (proL); total length (ToL).

### ﻿Taxonomic account

**Phylum Hemichordata**

**Class Enteropneusta**

**Family Ptychoderidae Spengel, 1893**

**Genus *Glossobalanus* Spengel, 1901**

#### ﻿ Glossobalanus weii sp. nov.

Taxon classificationAnimaliaEnteropneustaPtychoderidae

30C9E66B-3D95-5067-AF2C-B8DA9CC5AC32

https://zoobank.org/BD53EAB7-7B4C-400C-A441-80CF57B7F22D

Fig. 2

##### Material examined.

**Figure 2.**
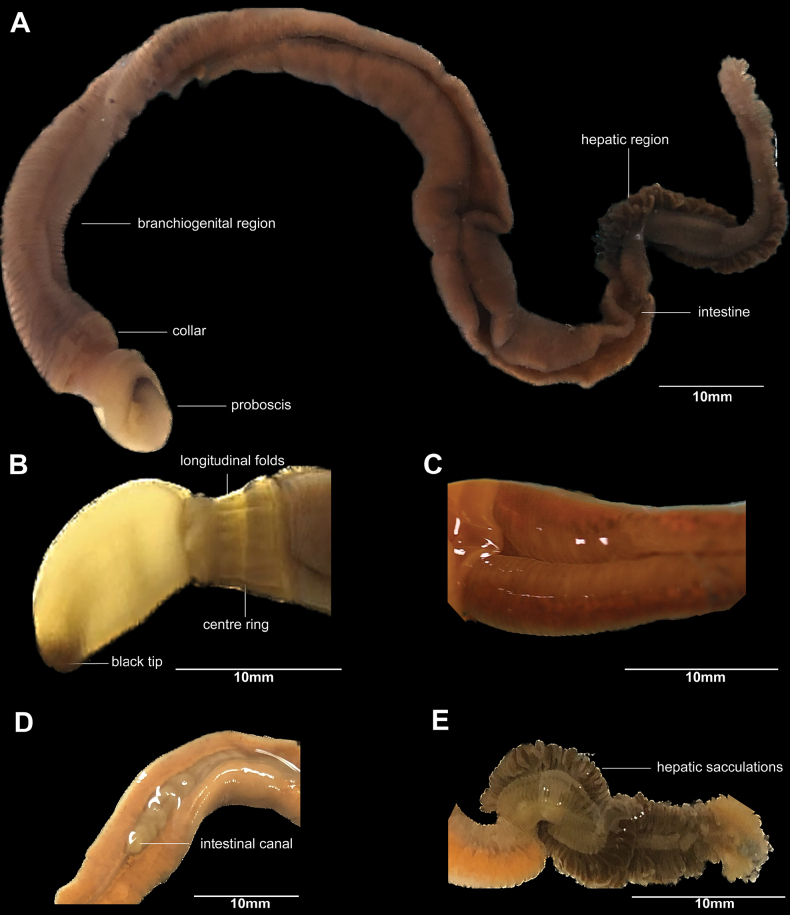
External form of *Glossobalanusweii* sp. nov. **A** whole body of female GML-23021883041 under natural conditions **B** proboscis and collar of paratype male GML-23021883042 **C** branchial region dorsal side photo of paratype male GML-23021883042 **D** intestinal canal containing food **E** hepatic region photo of paratype male GML-23021883042.

***Holotype*.**GML-23021883041 (Fig. 2A), adult female, collected from Toudong Village, Xinying Town, Lingao County, Danzhou City, Hainan Province, China (19°52.2035'N, 109°32.1094'E), collected by Xianan Fu, Weijian Guo and Wenliang Zhou on 23 Feb 2023 (GML). ***Paratypes*.**GML-23021883042 (Fig. 2B–D), adult male, same collection information as holotype. GML-23070380011, adult male, same location information as holotype, collected by Xianan Fu and Zhongwen Ding on 4 Jul 2023 (GML).

##### Diagnosis.

**Figure 3.**
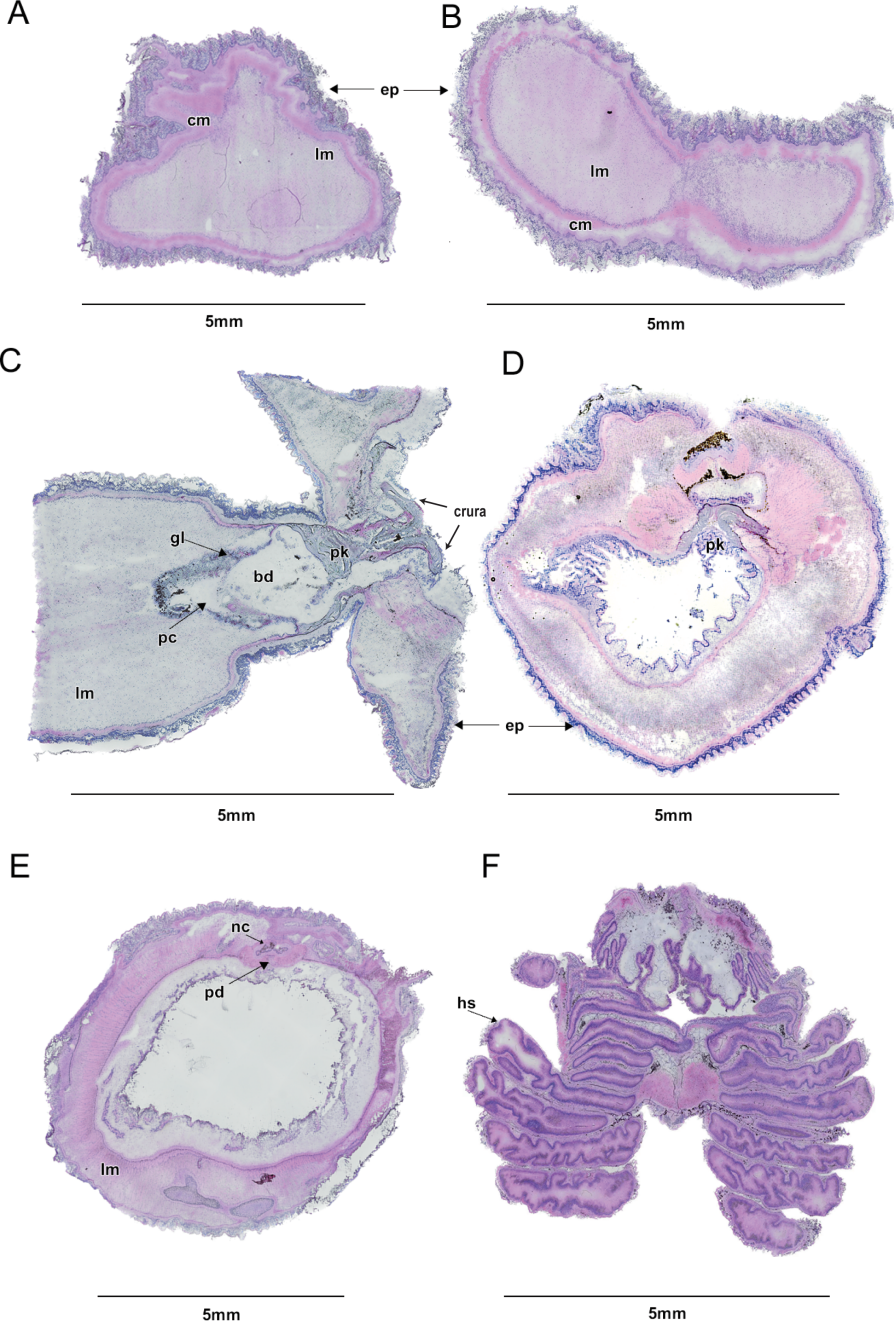
Histological characteristics of *Glossobalanusweii* sp. nov. **A** longitudinal section through the anterior proboscis **B** transverse section through the anterior proboscis **C** frontal section through the posterior proboscis and collar **D** transverse section through the anterior collar **E** transverse section through the posterior collar **F** transverse section through the hepatic region. Abbreviations: buccal diverticulum (bd); circular musculature (cm); epidermis (ep); glomerulus (gl); hepatic sacculations (hs); longitudinal musculature (lm); nerve cord (nc); proboscis cavity (pc); perihaemal diverticula (pd); proboscis skeleton (pk).

**Figure 4.**
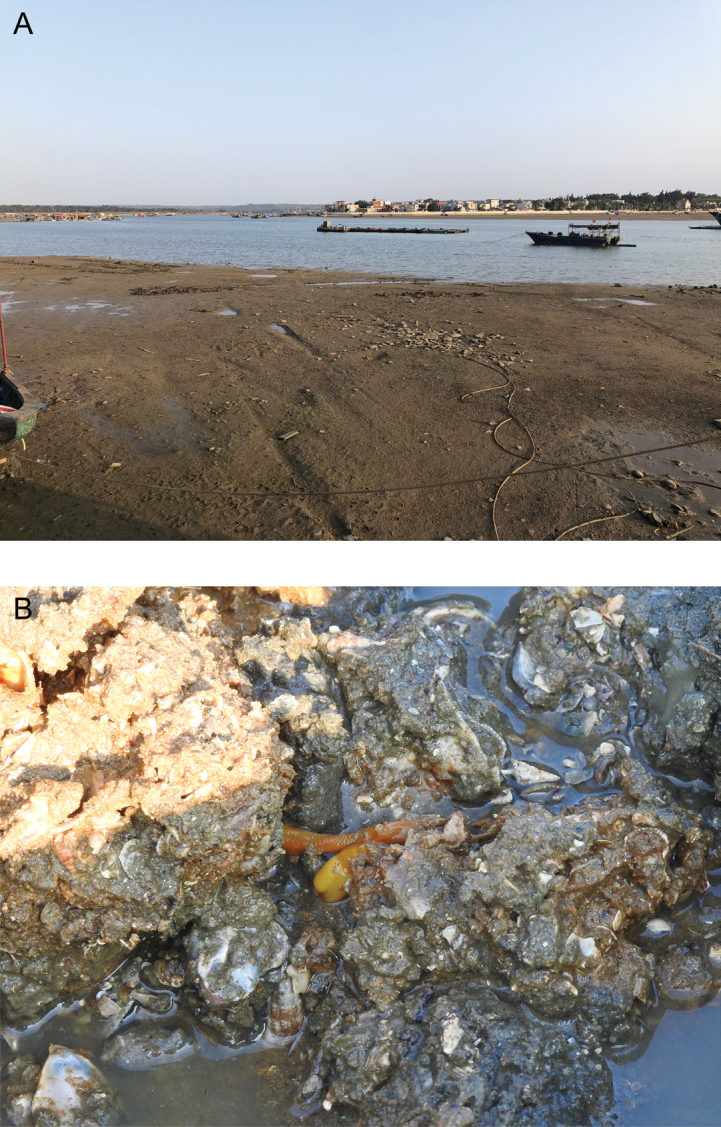
Habitat of *Glossobalanusweii* sp. nov. **A** the intertidal zone habitats at the type locality **B** the humic mudflats intertidal zone with gravelly and shell carcasses substrates feature.

**Figure 5.**
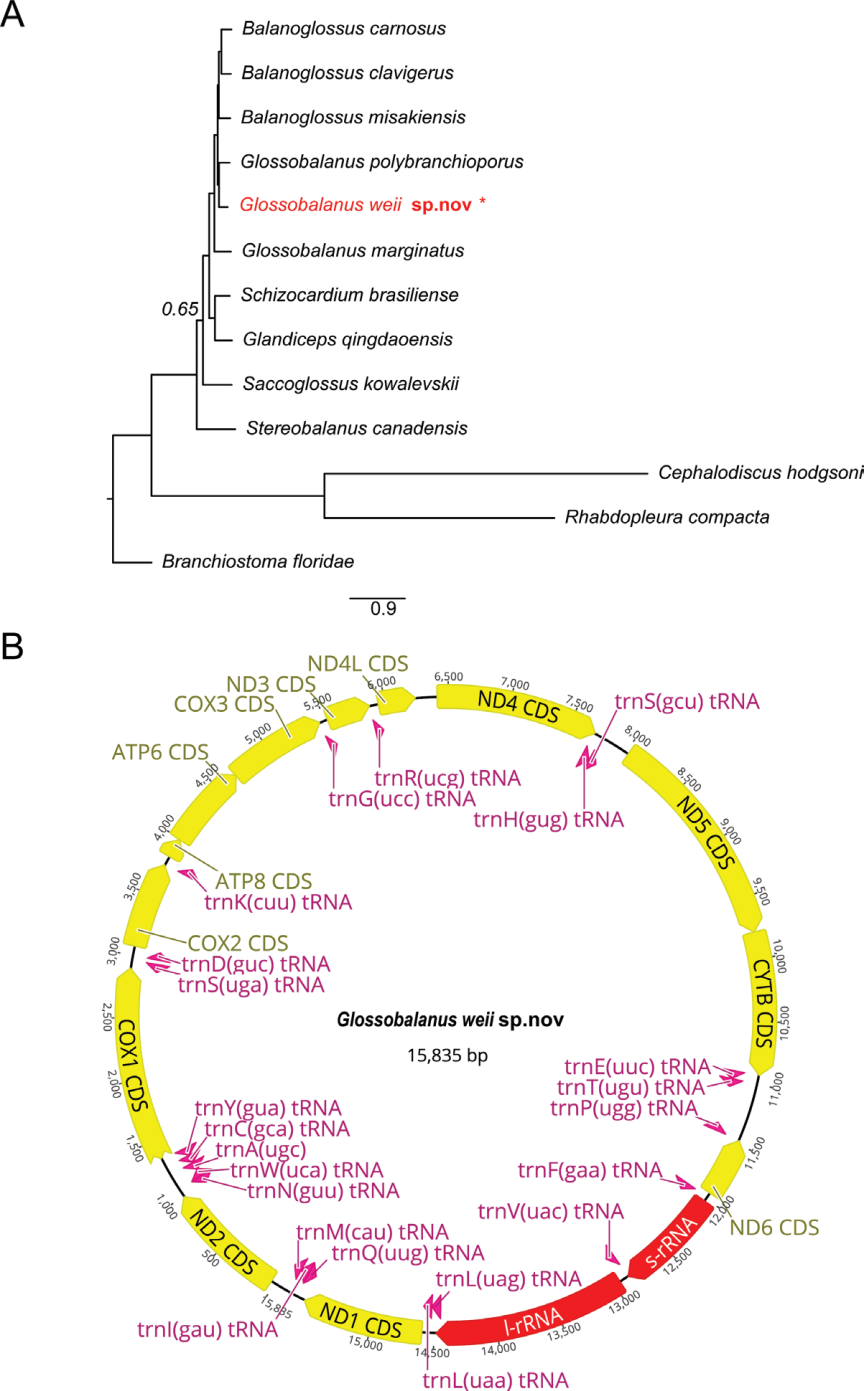
Phylogenetic analyses using BI based on 13 protein-coding genes in the mitochondrion of species of *Glossobalanus***A** the new species formed a distinct sister clade to *G.polybranchioporus.* The posterior probability of the node<1 is shown on the phylogenetic tree **B** map of the assembled and annotated *G.weii* sp. nov. mitochondrial genome.

*Glossobalanusweii* sp. nov. can be differentiated from closely related species by a combination of the following characters: (1) large worms with adult body size of more than 150 mm (Fig. 2A); (2) the proboscis is twice as long as the collar (Fig. 2B); (3) a dark spot on the tip of the proboscis (Fig. 2A, B); (4) a rather broad posterior collar margin and the dorsal margin of the collar is shorter than the ventral margin (Fig. 2B); (5) the collar is covered with longitudinal pleats bound tightly with a ring in the center (Fig. 2B); and (6) a slender brown hepatic region with plump sacculations (Fig. 2E).

##### Description.

Body of the holotype as in Fig. 2. The worm bodies are fragile and easily broken. When placed on a flat surface or photographic canvas they shrink severely and break and it is necessary to examine and measure them in an aqueous or similar solution. The complete living specimen is 176 mm long. There is a dark spot on the tip of the proboscis. The proboscis can stretch freely from 11 to 16 mm. The collar has a number of longitudinal folds with a pale-colored ring in the middle, and on the back, the collar has a raised broad edge forming another ring band (Fig. 2B). The dorsal collar length is 4.7 mm, and ventral collar length is 5.7 mm in the vertical direction; the width is 5–9 mm in the transverse direction. The anterior margin of the collar encircles the base of the proboscis, and there is a conspicuous mouth in the center of the ventral surface of the anterior end at the junction with the proboscis. Trunk is sub-cylindrical and divided into branchiogenital region, hepatic region, and intestinal region. The branchiogenital region is 105 mm long and 6.2 mm in transverse diameter, sub-cylindrical and slightly furrowed. There are 40 pairs of small gill pores that are invisible without magnification. The beginning of the branchiogenital region is connected with the posterior margin of the collar and covers the anterior part of the gill region (Fig. 2C). The slender intestinal canal extends from branchiogenital region through the hepatic region. The digestive tract narrows before the hepatic region (Fig. 2D). Hepatic region length is 24–26 mm with about 60 pairs of mature sacculations (Fig. 2E). The arrangement of the hepatic sacculations is plumper and sparser at the anterior, and progressively tighter and neater at the posterior end.

##### Coloration.

In fresh specimens, the body is generally brownish yellow, fading to pale yellow or pale tan in the proboscis and has a black tip. There is a pale, thin ring in the middle of the collar, and a darker colored annular band connects the collar and branchiogenital region of the trunk (Fig. 2B). The branchiogenital region is yellowish brown in females and pale yellow or honey yellow in males. The anterior portion of the hepatic trunk is dark brown and fading to pale brown on the posterior portion (Fig. 2E). The preserved specimens faded to a dull yellow. The hepatic region turned grey after fixation in alcohol.

##### Anatomical and histological characters.

The circular musculature and the epidermis of the proboscis have equal thickness around 0.3 mm (Fig. 3A, B). The proboscis is filled with slender muscles and the circular muscle is outside the longitudinal musculature (Fig. 3A, B). The proboscis cavity is small and may be missed in the transverse section (Fig. 3C). The buccal diverticulum was integrated with the pericardial coelom and surrounded by glomerulus (Fig. 3C). The proboscis bifurcates at the anterior collar into two crura at more than 180° (Fig. 3C). The proboscis has the appearance of a butterfly extending into the median of the collar on the dorsal side (Fig. 3D). The flattened nerve cord is surrounded by the perihemal diverticula (Fig. 3E). The hepatic sacculations are neatly arranged and symmetrical overall (Fig. 3F).

##### Natural history and distribution.

*Glossobalanusweii* sp. nov. is currently only known from the inlet of Toudong village of Danzhou, Hainan Province, China, in the Beibu Gulf (Fig. 1). The new species inhabits humic mudflat intertidal zones with gravelly and shell carcass substrates and is a burrowing filter-feeder (Fig. 4).

##### Etymology.

This species is named after Fuwen Wei to commend his contributions to Zoology and Conservation biology. We propose the common name “魏氏舌形虫” in Chinese.

##### Genetic results.

The validity of *G.weii* sp. nov. was supported by molecular phylogenetic analysis based on the aligned DNA sequences of 13 mitochondrional protein-coding genes (Fig. 5). Bayesian phylogenetic analyses revealed that the specimens form an independent clade with robust support and are closest to *G.polybranchioporus*. The uncorrected pair-wise sequence divergence between the new species and the other recognized relatives was > 25% (Table 3).

**Table 3.** Estimates of evolutionary divergence between hemichordates with whole mitochondrial genome.

Species	1	2	3	4	5	6	7	8	9	10	11
*Glossobalanusweii* sp. nov.											
*Glossobalanuspolybranchioporus*	0.255										
*Balanoglossusclavigerus*	0.267	0.285									
*Balanoglossusmisakiensis*	0.274	0.281	0.279								
*Balanoglossus.carnosus*	0.276	0.281	0.258	0.287							
*Glossobalanus.marginatus*	0.332	0.334	0.352	0.349	0.361						
*Schizocardiumbrasiliense*	0.377	0.388	0.401	0.402	0.405	0.407					
*Glandicepsqingdaoensis*	0.392	0.407	0.402	0.414	0.411	0.419	0.348				
*Saccoglossuskowalevskii*	0.514	0.516	0.550	0.509	0.547	0.522	0.533	0.546			
*Stereobalanuscanadensis*	0.461	0.464	0.469	0.453	0.471	0.478	0.447	0.476	0.531		
*Rhabdopleuracompacta*	1.091	1.053	1.065	1.039	1.063	1.070	1.013	1.060	1.021	0.953	
*Cephalodiscushodgsoni*	1.116	1.080	1.107	1.092	1.096	1.083	1.047	1.093	1.074	1.042	0.815

The number of base substitutions per site from between sequences are shown. Analyses were conducted using the Maximum Composite Likelihood model. This analysis involved 13 nucleotide sequences. All ambiguous positions were removed for each sequence pair (pairwise deletion option). Evolutionary analyses were conducted in MEGA11 (Tamura et al. 2021).

The complete mitogenome of *G.weii* sp. nov. is 15,835 bp long with a base composition of 15.8% G, 27.0% A, 25.4% T, and 31.7% C. It encoded 37 genes including 13 protein-coding genes, 22 tRNA genes, 2 rRNA genes, and a control region (Fig. 5B). All these genes were encoded on the heavy strand, except for the ND6 protein-coding gene and seven tRNA genes (tRNA-Ala, tRNA-Cys, tRNA-Asn, tRNA-Pro, tRNA-Gln, tRNA-Ser and tRNA-Tyr) which were located on the light strand.

Combined with the morphological comparison and phylogenetic data, we confirm that the specimens from Danzhou, Hainan Island represent a new species of the genus *Glossobalanus*.

### ﻿Key to the eight species of Enteropneusta in China

**Table:** 

1	Hepatic sacculations present; branchiogenital region highly developed to two dorsolateral rows of gill pores and serial gonads houses	**2**
–	Hepatic sacculations absent; branchiogenital region not well developed	**7**
2	Gill apertures broad; gonads only on the ventral of lateral septum	***Ptychoderaflava***
–	Gill apertures narrow; gonads on both the ventral and dorsal of lateral septum	**3**
3	Well-developed genital wings	**4**
–	Dorsolateral genital ridges	**5**
4	Proboscis length equal in collar; genital wing initiation separated from posterior margin of the collar by a small segment at the center of the dorsal surface, hepatic sacculations without serrated margins	***Balanoglossusmisakiensis***
–	Collar longer than proboscis, genital wing tip joined to posterior margin of collar; hepatic sacculations with serrated margins	***Balanoglossuscarnosus***
5	Genital ridges beginning at end of gill area, short and not prominent	***Glossobalanusmortenseni***
–	Genital ridges begin at the anterior end of the gill area, long and prominent	**6**
6	Proboscis length is less than twice of the collar; gill pores 130–160	***Glossobalanuspolybranchioporus***
–	Proboscis length is more than twice that of the collar; gill pores 30–60	***Glossobalanusweii* sp. nov.**
7	Proboscis 3× collar length; proboscis cavity separated completely by the median dorsal-ventral septum	***Glandicepsqingdaoensis***
–	Proboscis 4–9× collar length; proboscis cavity not separated completely by the dorsal-ventral septum	***Saccoglossushwangtauensis***

## ﻿Discussion

**Figure 6.**
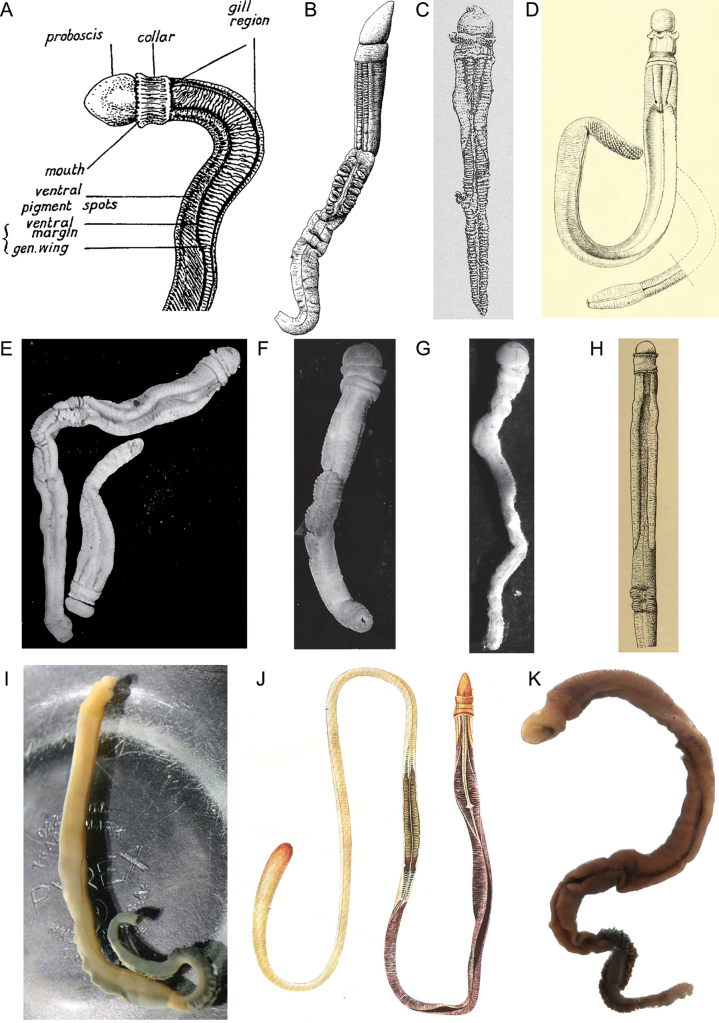
External morphology of genus *Glossobalanus* species **A***G.marginatus* in Meek (1922)**B***G.mortenseni* in van der Horst (1932)**C***G.crozieri* in van der Horst (1924)**D***G.ruficollis* in Willey (1899)**E***G.barnharti* in Cameron and Ostiguy (2013)**F***G.hartmanae* in Cameron and Ostiguy (2013)**G***G.minutus* in Cameron and Ostiguy (2013)**H***G.alatus* in van der Horst (1940)**I***G.berkeleyi.* Photo from Swalla and van der Land (2024). Hemichordata World Database **J***G.polybranchioporus* in Tchang and Liang (1965)**K***G.weii* in this article.

All the specimens of the new species were morphologically consistent with the other recognized species of the genus *Glossobalanus* (Fig. 6). In addition to differences in morphological indicators such as the total length, the branchiogenital region and the collar length, the new species can be distinguished by a black tip to the proboscis, a proboscis that is twice the length of the collar, a broad posterior collar margin and string brown hepatic sacculations. *Glossobalanuspolybranchioporus* is the only other *Glossobalanus* species also found in China. The total body length of the new species is 176–196 mm, half the body length of *G.polybranchioporus* (352–613 mm; Tchang and Liang 1965). Meanwhile, the number of branchial pores is around 40, much less than *G.polybranchioporus* (130–160 mm). Our histological analysis also observed that the morphological characteristics of new species is different from other *Glossobalanus* species in a rather small proboscis cavity and > 180° proboscis skeleton bifurcation. The polygenic molecular genetic analysis show that the new species is clustered together with *G.polybranchioporus.* One surprise was that another species, *G.marginatus*, did not cluster in the same clade. This may require a higher level of taxonomic determination, or it may be for other reasons we do not yet know. More attention and research work needs to be devoted to this in the future. Integrating external morphology, histology, and molecular analysis, we present the specimens from Danzhou city, Hainan Island, as a new species of *Glossobalanus* (Enteropneusta).

To better understand the living habits and distribution of *G.weii* sp. nov. in the field, we also conducted field investigation in a wide range. *Glossobalanusweii* sp. nov. usually inhabits benthic gravelly mud, rich in crustacean shells (Fig. 4B), where it occurs over a limited area in discrete patches. This species of acorn worms may require a certain sand quality in their habitat. For example, under experimental conditions, the worms will not burrow in the fine sand from which the shell residue is manually removed, which may greatly limit their movement and ability to avoid enemies. Construction of coastal features with a large amount of fine sand will inevitably adversely affect the survival of this species. On the other hand, according to the current investigation results, the distribution range of *G.weii* sp. nov. is extremely narrow, and the known distribution of *G.polybranchioporus* is 2000 km from its related species (Fig. 1). It is possible that further surveys will extend the range, or that the patchy distribution of these species indicates the demise of an ancient and declining group (Cameron et al. 2010). However, it is unfortunate that the narrow distribution of two acorn worm species overlaps extensively with infrastructure projects development in the coastal zone, which have encroached upon their habitats in recent years. In the future, it is worth thinking about how we and hemichordates will live together.

More than 130 species of hemichordate have been recorded worldwide, and most live in intertidal mudflats or deep-sea environments (Tassia et al. 2016). There is a serious lack of understanding of the distribution and habits of extant hemichordates, mainly due to the lack of public attention, specialized systematic investigation as well as scientific research by researchers on this species group. Most of the known species and even genera have been described only once. In addition, many species may already be endangered or extinct before being formally described. In China, hemichordates have received little attention with only seven species of Enteropneusta being recorded (Liang 1984; An and Li 2005), and their species richness may be greatly underestimated. With this discovery, we aim to shed light on conservation efforts for Chinese hemichordate species. Either way, it is necessary for us to pay more attention to this important group of evolutionary nodes in the future, especially to carry out more detailed studies on the classification and endangered status of them.

## Supplementary Material

XML Treatment for Glossobalanus weii

## ﻿Additional information

### Author ORCIDs

Xianan Fu https://orcid.org/0000-0001-9359-6314

Wenliang Zhou https://orcid.org/0000-0002-1240-8842

Fuwen Wei https://orcid.org/0000-0002-5233-1288

### Data availability

All of the data that support the findings of this study are available in the main text.
